# Converging Epidemics: A Narrative Review of Tuberculosis (TB) and Human Immunodeficiency Virus (HIV) Coinfection

**DOI:** 10.7759/cureus.47624

**Published:** 2023-10-25

**Authors:** Asmi Bhatt, Zahiruddin Quazi Syed, Harshit Singh

**Affiliations:** 1 Community Medicine, Jawaharlal Nehru Medical College, Datta Meghe Institute of Higher Education and Research, Wardha, IND; 2 Pathology, Jawaharlal Nehru Medical College, Datta Meghe Institute of Higher Education and Research, Wardha, IND

**Keywords:** prevention, treatment, diagnosis, human immunodeficiency virus, tuberculosis, coinfection

## Abstract

Tuberculosis (TB), primarily caused by *Mycobacterium tuberculosis* (MTB), remains a leading cause of mortality in individuals living with human immunodeficiency virus (HIV) infection, posing a significant strain on healthcare systems. Coinfection of HIV and TB results in a mutually advantageous relationship that accelerates the progression of both diseases. TB is a major contributor to mortality in individuals with HIV. However, diagnosing coinfected individuals is challenging due to the prevalence of extrapulmonary TB and smear-negative disease. Over the past decade, significant progress has been made in the fight against TB, thanks to advances in molecular techniques. Yet, these molecular diagnostic assays remain inaccessible to many individuals coinfected with HIV and TB due to their high cost. To expedite treatment and reduce transmission, it is crucial to integrate HIV and TB control programs more closely, thereby minimizing diagnostic delays and enhancing early case detection.

This review aims to provide a comprehensive overview of the current state of knowledge regarding the interplay between HIV and TB. It highlights recent developments in sensitive and rapid TB diagnostic tests, cutting-edge preventive strategies, and the screening of individuals coinfected with both HIV and TB. The objectives of this review are to shed light on the complex relationship between these two diseases and to emphasize the importance of integrated efforts in combating their impact on public health.

## Introduction and background

Within the realm of infectious diseases, the human immunodeficiency virus (HIV/AIDS) and tuberculosis (TB) have emerged as formidable adversaries, imposing a staggering burden, particularly on nations grappling with limited healthcare resources. The intricate interplay of these two maladies within the human host creates a synergistic dyad, expediently accelerating the erosion of the immune system. Left unchecked, this lethal partnership elevates the specter of premature mortality. In the annals of public health, the year 2020 bore witness to a sobering reality, with a reported 9.9 million novel TB cases and a distressing toll of 1.5 million TB-related deaths [1]. Of profound significance is the observation that among individuals bearing the dual burden of HIV and TB, the latter emerges as the predominant cause of infectious fatalities. This dual burden strains healthcare systems in multiple ways. Firstly, it significantly increases mortality rates, as TB and HIV can synergistically worsen each other's effects. Diagnosis can be complicated, often requiring specialized testing and expertise due to atypical symptoms and compromised immune systems in HIV-positive individuals. Treatment is extended and complex, potentially leading to medication interactions and side effects that demand meticulous monitoring.

The emergence of drug-resistant TB (DR-TB) further complicates the management landscape. Preventive therapy is necessary to reduce TB risk in HIV-positive individuals, placing additional strain on healthcare systems. Public health interventions, including contact tracing and infection control, are essential. Overall, this dual epidemic diverts resources and poses substantial challenges for healthcare systems. This assertion is validated by meticulous postmortem examinations, encompassing both adult and pediatric cohorts afflicted by HIV, thereby underscoring the formidable diagnostic challenges microbiologists confront when endeavoring to unveil the insidious presence of TB amidst the complex backdrop of HIV coinfection [2]. In the realm of diagnostics, the World Health Organization (WHO) champions two stalwart methodologies: smear microscopy and mycobacterial culture, as indispensable tools for the detection of this vexing coinfection. Notably, while direct microscopy offers a swift and cost-effective means to identify tuberculosis, it regrettably falters in sensitivity when juxtaposed with more sophisticated counterparts [3]. Nevertheless, the contemporary medical landscape is marked by the advent of molecular diagnostic procedures, poised to surmount the limitations encumbering traditional laboratory techniques, ushering in a new era of precision and accuracy [4].

Diagnosing individuals coinfected with HIV and TB, particularly cases of extrapulmonary TB (EPTB) and smear-negative disease, poses several formidable challenges. In EPTB, the disease affects organs outside the lungs, making symptoms less specific and the diagnosis more complex. Furthermore, HIV can mask typical TB symptoms, leading to atypical clinical presentations. Smear-negative TB cases, where acid-fast bacilli are not detectable in sputum, require more sophisticated and costly diagnostic techniques like culture or molecular tests, which may not be readily available in resource-limited settings. The compromised immune system in HIV-positive individuals can yield false-negative results in traditional TB tests, necessitating more specialized and sensitive assays. These diagnostic challenges delay the timely and accurate identification of TB in coinfected patients, hampering early intervention and increasing the risk of disease progression and transmission, underscoring the need for improved diagnostic tools and strategies in such cases.

Below the surface of this intricate interplay between HIV and TB lies a realm of enigmatic processes that subvert the host's immune defenses. The coinfection of HIV and TB creates a mutually disadvantageous relationship for the individuals affected. HIV weakens the immune system, making those with the virus more susceptible to TB infection, which, in turn, can lead to the activation of latent TB. This mutual interaction accelerates the progression of both diseases. TB infection can cause a rapid increase in HIV replication, worsening the progression of HIV. Simultaneously, the presence of HIV significantly increases the risk of active TB development in those with latent TB infection. As a result, coinfected individuals experience more severe forms of both diseases, with higher mortality rates. This intertwined relationship underscores the importance of addressing both infections comprehensively to effectively manage and control their impact on affected individuals and public health. HIV infection emerges as the paramount predisposing factor for the resurgence of *Mycobacterium tuberculosis *(MTB), imparting a formidable 20-fold augmented risk of TB reactivation [5]. Paradoxically, TB reciprocates, fostering an environment conducive to heightened susceptibility to HIV infection in individuals [6]. The confluence of genetic variants and intrinsic immune modulations is increasingly recognized as a pivotal determinant of susceptibility to both HIV and TB, illuminating the intricate tapestry of host-pathogen interactions [7].

This narrative review embarks on an odyssey through the labyrinthine interplay of HIV and TB, elucidating their complex dynamics, diagnostic challenges, and the intriguing interweaving of host genetics and immunity. The primary objectives of this review are to illuminate the intricate interplay between HIV and TB and underscore the critical significance of integrated efforts in mitigating their impact on public health. By examining the complex relationship between these two diseases, the review seeks to provide a comprehensive understanding of how they exacerbate each other's effects, contributing to higher mortality rates. It aims to emphasize the need for integrated approaches in prevention, diagnosis, and treatment, as siloed strategies are inadequate in addressing coinfection challenges. The review ultimately advocates for a holistic, synergistic approach to combating the dual burden of HIV and TB, enhancing the effectiveness of public health interventions, and thereby reducing the overall impact of these diseases on affected individuals and communities.

## Review

Methodology

**Figure 1:**
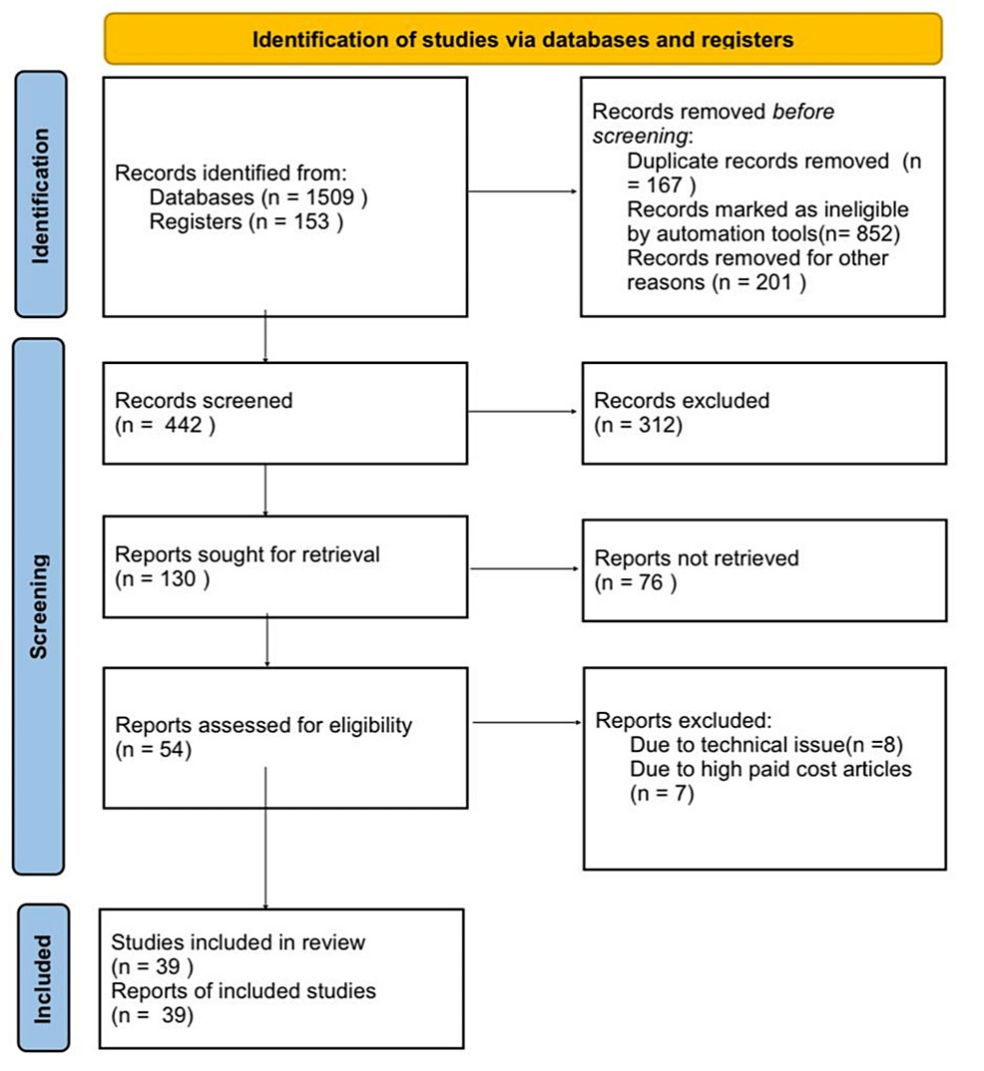
Search strategy employed for this review

Our investigation involved using databases such as PubMed to search for terms like 'tuberculosis,' 'Human Immunodeficiency Virus,' and 'antiretroviral therapy.' We specifically limited our search to results in the English language. In cases where multiple reports of the same study were identified, we selected the most recent one. Our primary focus was on review papers that presented recent findings and insights. Figure 1 shows the search strategy employed.

Epidemiology

TB remains a paramount cause of mortality stemming from infectious agents on a global scale. It is pertinent to note that sexual transmission accounts for roughly 80% of all documented cases of HIV infections worldwide. Additionally, approximately 10% of newly acquired HIV infections are observed among individuals who engage in injection drug use [8]. For individuals without HIV but harboring latent tuberculosis infection (LTBI), the probability of this infection transitioning into an active state over their lifetime falls within the range of 5-10%. In stark contrast, HIV-positive individuals face a significantly heightened risk, with annual rates of LTBI progressing to active TB ranging from 3% to 16% [9].

Notably, the augmented susceptibility to TB following HIV acquisition manifests promptly, even in the presence of elevated CD4 cell counts. HIV presence substantially amplifies the likelihood of transitioning from latent to active TB infection. According to the World Health Organization (WHO), individuals living with HIV face a staggering 26-fold increased probability of progressing to active TB disease in comparison to their HIV-negative counterparts [10].

Pathogenesis

TB poses a heightened risk to individuals with compromised immune systems, particularly those with HIV infection. TB is primarily caused by MTB, and occasionally other strains within the MTB complex [11]. When these bacteria enter the respiratory tract, they invade macrophages along with CD4+ T-lymphocytes. These CD4+ T-lymphocytes produce immune factors such as interferon-gamma (IFN-gamma), interleukin-2, macrophage colony-stimulating factor, and tumor necrosis factor-alpha (TNF-alpha). These factors activate macrophages and cytotoxic cells to combat intracellular MTB growth [12]. However, TB develops when the immune response, which triggers granuloma formation, is insufficient to halt mycobacterial proliferation. In HIV infection, decreased CD4+ T-lymphocytes significantly reduce IFN-gamma production, elevating the risk of MTB reactivation.

HIV-positive individuals face a considerably higher annual risk of TB compared to immunocompetent adults. The decline in CD4+ T cells in HIV infection contributes to the increased likelihood of latent TB reactivation and susceptibility to new MTB infections [13]. Several mechanisms facilitate TB infection in the presence of HIV, including upregulated MTB entry receptors on macrophages, manipulation of macrophage bactericidal pathways by HIV, disrupted immune responses, and alteration of granuloma integrity [14]. These interactions lead to a higher risk of active pulmonary TB and extrapulmonary transmission in coinfected individuals. HIV impacts TB progression by increasing the likelihood of latent TB transitioning to active TB, particularly in regions with high disease prevalence. EPTB also poses an increased risk in HIV-infected individuals due to CD4 T cell depletion and other factors [15-17]. Additionally, a mechanism believed to contribute to the heightened risk of TB reactivation in HIV-infected patients with latent TB involves a decline in the number of these cells, resulting in an inability to effectively manage and sustain granulomas in the host body. Apoptosis of infected macrophages acts as a significant immune response against TB infection in terms of innate immunity. A recent case-control study conducted in Brazil uncovered a distinct association between specific inflammasome gene variants, particularly CARD8 mutations, and the progression of TB infection in HIV-positive individuals [18].

In summary, TB susceptibility is significantly heightened in individuals with compromised immune systems, particularly those with HIV. Complex interactions between HIV and TB involve various immune factors and mechanisms, leading to a higher risk of TB reactivation, progression, and extrapulmonary manifestations in coinfected individuals.

**Figure 2:**
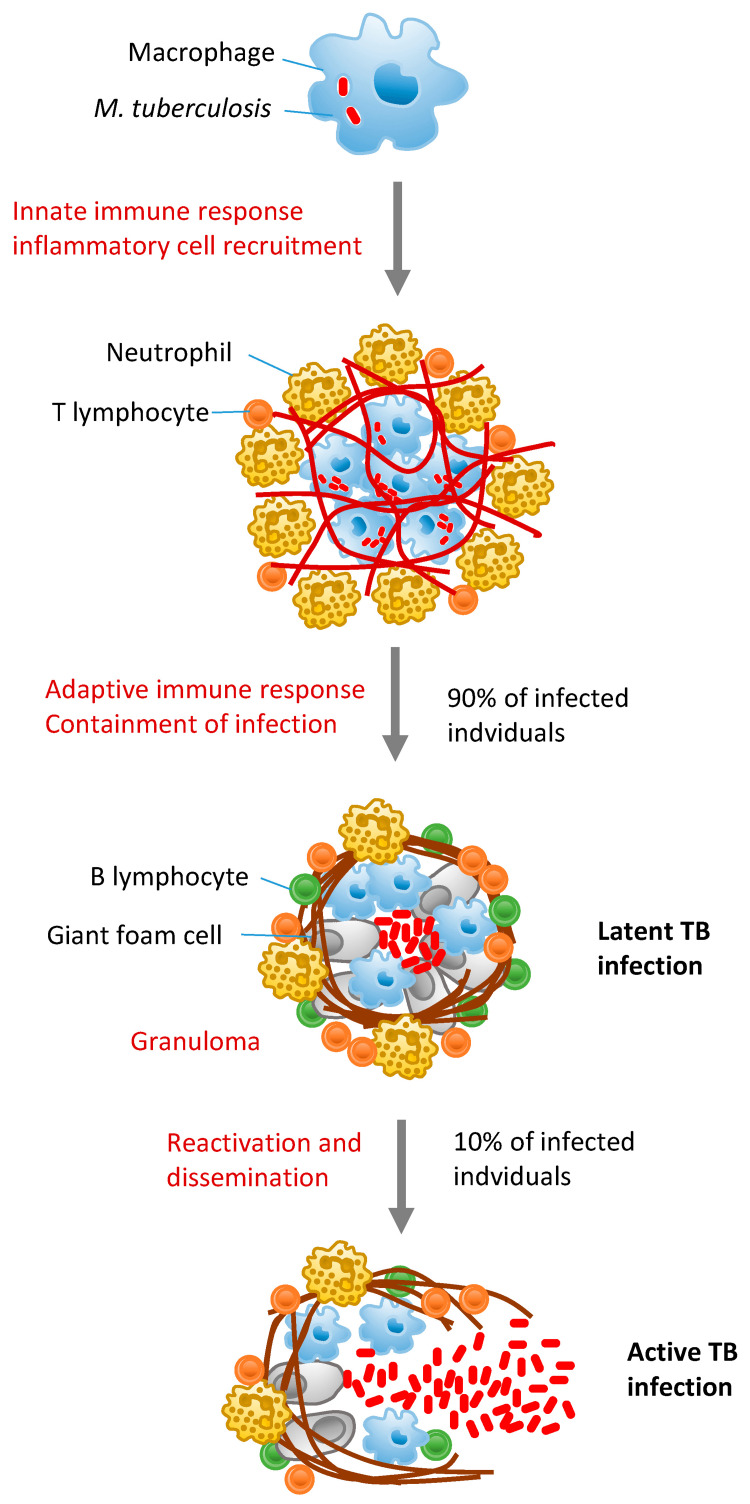
Figure depicting TB disease progression and pathophysiology Inhalation of bacteria-carrying droplets and alveolar macrophage absorption starts MTB infection. The earliest phase of infection involves innate immune responses and lung inflammatory cell recruitment. Antigen-specific T lymphocytes are primed and expanded by bacterial propagation to the draining lymph node. Activated macrophages, neutrophils, T cells, and B cells in the lung create granulomas with latent MTB MTB: *Mycobacterium tuberculosis* Image under CC-BY license contributed by A. Sinigaglia. Source: [18]

Figure 2 below depicts TB disease progression and pathophysiology.

HIV and TB Synergy Mechanism

HIV-infected individuals are more vulnerable to TB due to several key mechanisms. Primarily, HIV weakens the immune system, particularly CD4 T cell function, making the body less capable of containing LTBI or fighting active TB disease. Secondly, HIV disrupts the granuloma formation in the lungs, which is the body's natural defense mechanism against TB. Additionally, HIV increases the risk of reactivation of LTBI, making individuals more likely to progress to active TB. In recent years, our understanding of these mechanisms has improved thanks to research into the intricate immune interactions between HIV and TB. This includes a deeper understanding of immune cell dysfunction, the impact of viral load, and the role of specific cytokines and chemokines. These insights have led to the development of more targeted approaches to managing coinfection and improving treatment outcomes for affected individuals.

Diagnosis

Early detection of LTBI is crucial for all HIV-positive individuals upon HIV diagnosis. Patients with HIV-TB coinfection often present with atypical clinical manifestations and imaging findings due to their compromised immune systems and lower bacterial load in respiratory secretions. Traditional TB diagnostic techniques, such as sputum smear, sputum culture, tuberculin skin tests, and interferon-release tests, have shown suboptimal sensitivity in detecting the disease. Disturbingly, autopsies in South Africa revealed that approximately 45.8% of HIV-infected individuals succumbed to TB without ever receiving a diagnosis [19]. This suggests that the prevalence of TB among HIV-positive individuals may be underestimated. The integration of HIV and TB control programs is crucial for minimizing diagnostic delays and enhancing early case detection among coinfected individuals. When these programs work together, healthcare systems can implement joint screening and testing protocols that identify both HIV and TB in a single visit, streamlining the diagnostic process. This integrated approach also promotes more comprehensive healthcare, ensuring that individuals receive appropriate treatment for both diseases promptly. Moreover, it enables healthcare providers to address challenges such as drug interactions and side effects more effectively. By unifying efforts, healthcare systems can better target coinfected populations, reduce diagnostic delays, and ultimately improve the early detection and management of both HIV and TB, enhancing the overall health outcomes in coinfected individuals.

Diagnostic Test for HIV

The CD4 count assesses the health of our immune system by counting the number of CD4 T cells per milliliters (cells/mm^3^) of blood. The general categories for CD4 counts are as follows - immune suppression: 200-499 cells per milliliter; standard value: 500 cells per mm^3^ or greater; AIDS: less than 200 cells/mm^3^.

Diagnostic Test in HIV-Associated TB

Clinical screening algorithms for TB in individuals with HIV are essential for early detection and treatment. According to WHO, TB screening should begin immediately upon identifying an HIV infection before starting antiretroviral medication and should be conducted regularly during follow-up care. Effective TB screening requires a comprehensive discussion of symptoms beyond just a persistent cough. Among the various conditions considered, recent weight loss, night sweats, fever, or cough were found to be the most effective indicators. This screening guideline has a diagnostic sensitivity of 79%, which increases to 90% in clinical conditions. However, its specificity is 50%, and it exhibits a substantial negative predictive value for both high and low CD4 levels and different TB frequencies [20].

Pulmonary TB typically manifests in patients with relatively intact immune function, characterized by a CD4 T cell count exceeding 200/mm. Symptoms include cough, sputum production, and less frequently, hemoptysis, thoracic discomfort, and dyspnea, observed in 70-93% of TB patients. Severely immunocompromised individuals with HIV tend to exhibit more frequent pulmonary basal involvement, tuberculous pneumonia, lymphadenopathies in the hilar or mediastinal regions, and miliary TB [21]. The association of pulmonary and extrapulmonary TB occurs in 9-40% of cases [22]. EPTB, especially in highly immunocompromised HIV patients, is common and can involve various organs. Reactivation of a dormant infection causes these symptoms, and CT scanning can help detect extrapulmonary lesions [23,24]. Persistent, unexplained fever is a significant symptom that may necessitate anti-tuberculous treatment. Recent advances in molecular techniques have significantly enhanced TB diagnosis, particularly in coinfected individuals. Nucleic acid amplification tests (NAATs), such as GeneXpert, offer rapid and highly sensitive detection of TB and drug-resistant strains. These tests are crucial for those coinfected with HIV, as they often have atypical TB presentations. Despite these advancements, accessibility remains a challenge. The cost of these assays, infrastructure requirements, and the need for trained personnel limit their availability in resource-constrained settings where TB and HIV are prevalent. To combat this, efforts are ongoing to make these diagnostic tools more affordable and widely accessible, ensuring that coinfected individuals, who often reside in vulnerable communities, can benefit from improved and timely TB diagnosis and treatment [24].

Smear Microscopy

When examining sputum smears under a microscope, acid-fast mycobacteria are visible in patients with TB infection. The most straightforward and effective method for detecting MTB is through light microscopy examination of Ziehl-Neelsen (ZN)-stained slides. MTB, an acid-fast bacillus, appears bright red when observed microscopically. In regions where TB is endemic, direct microscopy is currently favored due to its affordability, speed, and high specificity. However, it has a significant drawback - its sensitivity is poor and variable, ranging from 20% to 60% [25]. Fluorescent microscopy offers improved sensitivity and expedites the screening of multiple smears. However, it is a more expensive test. Nonetheless, transitioning to fluorescent light-emitting diode (LED) lighting instead of traditional fluorescent light sources can substantially reduce costs [15]. As a result, the primary method recommended by WHO for smear microscopy is the replacement of conventional fluorescent techniques with LED microscopy.

Growth-Based Detection

MTB culture offers significantly higher sensitivity compared to smear microscopy and allows for drug susceptibility testing and strain characterization. Solid media like Middle Brook or Lowenstein-Jenson (L-J) are traditionally used for culturing but can be slow, with growth often not visible for six to eight weeks after incubation. This delay in starting therapy can lead to poorer outcomes for patients with HIV-TB coinfection. Automated liquid culture systems are more efficient, detecting mycobacterial growth within one to two weeks by measuring carbon dioxide or oxygen consumption, using methods such as radiometry, fluorescence, colorimetry, pressure, or redox reagents like Alamar Blue-3. An affordable, non-commercial method for detecting microcolonies, cord development, and early drug resistance is the microscopic observation drug susceptibility (MODS) assay. It is more sensitive, provides quicker responses to culture positivity, and is cost-effective compared to the standard L-J medium. The FASTPlaque TB™ assay (Biotec Laboratories Ltd., Ipswich, UK), with a specificity of 98%, can detect MTB in 50-65% of smear-negative samples. These tests offer reasonably high accuracy when used with culture isolates. However, their sensitivity in the context of HIV-TB coinfection may be limited due to the potential for contamination [22].

Serological Detection of TB (Tuberculin Skin Test)

The significance of skin tests in diagnosing TB varies based on the patient's immune condition. A positive tuberculin test result and the development of a granulomatous tissue reaction depend on a functional Th1 cytokine response. In individuals with HIV, the tuberculin test yields a positive result in 30% of cases with a CD4 cell level of 200/mm^3^ and 50% in those with a CD4 cell level exceeding 200/mm^3^. The occurrence of tuberculous granulomas, which can range from 60% to 100%, depends on both the immunological status of the patient and the site of the sample [22,26].

Bacteriophage-based methods, such as the FASTPlaque TB™ assay, have been utilized for TB diagnosis. The FASTPlaque TB™ assay boasts a specificity of 98% and can detect mycobacteria in 50-65% of samples that are negative in smear tests. These assessments demonstrate notable accuracy, particularly when used in conjunction with culture isolates [27]. However, their sensitivity in the context of HIV-TB coinfection is limited, primarily due to an increased risk of contamination.

Treatment

Individuals with HIV and TB coinfection are advised to receive combined treatment for both conditions, regardless of their CD4+ cell count. This treatment involves the use of antiretroviral therapy (ART) alongside anti-tuberculosis treatment (ATT). However, there is an ongoing debate about when to initiate ART.

Commencing ART early offers numerous benefits, including reduced early mortality, lower relapse rates, prevention of drug resistance in ATT, and a decreased prevalence of HIV-related diseases, other than TB. However, there are also drawbacks to consider, such as potential drug interactions leading to inflammatory responses, cumulative toxicity from both ART and ATT, and other factors that may limit their use. In the context of ATT, the standard approach involves the use of four medications - isoniazid (H), rifampicin (R), pyrazinamide (Z), and ethambutol (E) - during a two-month intensive phase, followed by a four-month continuation phase featuring HRE. Regarding ART for HIV-TB coinfection, WHO guidelines recommend an initial treatment approach combining two nucleoside reverse transcriptase inhibitors (NRTIs) and one non-nucleoside reverse transcriptase inhibitor (NNRTI).

TB Treatment in HIV-Infected Individuals

The effectiveness of primary TB medications in eradicating MTB and preventing drug resistance varies. Isoniazid, the most potent antibacterial agent, targets metabolically active bacteria, resulting in the demise of over 90% within a week and reducing the risk of drug resistance. Rifampicin, another antibacterial medication, has strong sterilizing effects and can also hinder drug resistance. Pyrazinamide, primarily valued for sterilization, excels in eliminating bacilli within acidic environments like macrophages. Ethambutol is less effective than streptomycin but can act as a bactericidal agent at higher concentrations. HIV-infected individuals at higher risk of drug resistance may consider including ethambutol, streptomycin, or amikacin as supplementary medications during initial ATT.

Recent guidelines from the Centers for Disease Control and Prevention recommend a six-month TB treatment regimen using the same drug combination as for HIV-negative patients (rifampicin, isoniazid, ethambutol, and pyrazinamide) for HIV-positive individuals with drug-susceptible TB. For pulmonary TB, treatment may extend to nine months, shortened to four months if the culture turns negative early, provided there is a prompt clinical or bacteriological response. Prolonged treatment (9-12 months) is suggested for extra-thoracic or disseminated TB. For miliary, meningeal, or skeletal TB, treatment should last at least 12 months. Routine assessment of bacterial eradication in HIV-infected individuals should be performed when possible [28,29].

However, two studies have revealed contrasting findings: HIV patients treated with anti-TB medication for six months experienced higher recurrence rates than those receiving treatment for 9-12 months [30]. A concise overview of the anti-TB regimen applicable to HIV-coinfected patients is presented in Table 1.

**Table 1:** Anti-TB regimens for HIV coinfected patients* *Adapted from CDC guidelines [30]

Drug resistance	Patients with TB and HIV coinfection	Patients with only TB infection
None	HRZE for two months, HR for 4–7 months, or HZE plus rifabutin for two months; H plus rifabutin for 4–7 months	HRZE for two months, HR for four months
Isoniazid	RZE for 6-9 months or rifabutin + ZE for 6-9 months	RZE for six months
Rifampicin	HZE for 18-24 months or HZSE for two months; HZS for 7-10 months	HPE for 18-24 months

HIV Treatment in TB-Infected Patients

The availability of ART for managing HIV infection in resource-limited settings has improved considerably. However, a significant number of individuals in need of ART initiate treatment at an advanced stage, often already presenting with clinically severe TB when seeking medical care. For many coinfected patients, the simultaneous administration of ART and ATT is imperative, as it substantially enhances survival. Yet, this co-administration also poses numerous management challenges, including potential drug interactions, overlapping drug-related toxicities, and the emergence of tuberculosis-immune reconstitution inflammatory syndrome (TB-IRIS) [31].

Regardless of the CD4+ cell count, treatment for coinfected individuals is recommended due to the survival benefits associated with timely initiation of ART in all HIV-infected individuals, including those co-diagnosed with TB. This approach underscores the significance of early intervention, emphasizing the crucial role of ART in improving outcomes in this vulnerable population.

Highly Active Antiretroviral Therapy (HAART)

Highly active antiretroviral therapy (HAART) delivers significant benefits by gradually restoring specific CD4 cell immune responses directed against MTB [32]. However, this immune reconstitution, especially in the initial months of HAART, may transiently exacerbate existing TB symptoms or lead to the emergence of new clinical manifestations, symptoms, or radiographic findings [33]. In HIV-infected individuals concurrently managing TB and undergoing HAART, this phenomenon is recognized as TB-IRIS, documented in approximately 7% to 36% of cases [33]. Notably, these effects can be more pronounced when HAART is initiated closely following the commencement of TB treatment.

In scenarios involving NRTIs or NNRTIs, there is typically no need for dosage adjustments when co-administered with rifampicin, a cornerstone of ATT. However, it is imperative to avoid concurrent use of rifampicin with protease inhibitors (PIs) due to the potential elevation of hepatic enzyme levels, rendering them less effective, often leading to suboptimal outcomes. The standard regimen usually comprises two NRTI/NNRTI agents, an ATT regimen involving rifampicin, and a combination of all four drugs. Remarkably, among the PIs compatible with co-administration alongside rifampicin, saquinavir/ritonavir and lopinavir/ritonavir are notable options. Despite the higher cost involved, rifabutin presents distinct advantages when used in conjunction with PIs, effectively mitigating potential drug interactions and associated complications [32].

Complications of Antiretroviral Therapy

While ART plays a pivotal role in managing HIV and TB coinfection, it can sometimes trigger complications, of which TB-IRIS is noteworthy. TB-IRIS can manifest following the initiation of ART and falls into two categories: it may either manifest as a clinical presentation of previously undetected TB or result in the paradoxical deterioration of TB disease despite ongoing anti-TB treatment [34]. Typically, it emerges around three months after commencing ART, particularly in individuals with severe immune impairment. This phenomenon is often accompanied by a substantial decline in viral load and an increase in CD4 cell count. Treatment approaches for TB-IRIS vary, with non-inflammatory drugs employed in mild cases and steroids in moderate-to-severe instances, usually in conjunction with standard TB and HIV therapies. Importantly, most patients can continue ART without interruption. Challenges arise in managing TB and HIV concurrently due to the convergence of pharmaceutical toxicities, a substantial drug regimen, and potential drug interactions, thereby complicating the treatment landscape [34].

Primary and Secondary Prophylaxis for TB in HIV Coinfected Individuals

Primary prevention is paramount for individuals with HIV who exhibit a positive tuberculin skin test and have not previously undergone TB treatment. This holds true for those who have recently had close contact with individuals diagnosed with TB. For HIV-positive patients with LTBI, several recommended treatment strategies exist. Among these, a frequently advised approach is the administration of daily or twice-weekly isoniazid preventive treatment over a nine-month period, with no discernible interactions with HAART [35,36]. Currently, alternatives such as daily pyrazinamide and two months of either rifampicin or rifabutin are also recommended as preventive regimens [37]. The incidence of relapses is notably rare when anti-tuberculous medications are administered correctly. Consequently, supplementary TB prophylaxis is not typically recommended for individuals living with HIV [38,39]. However, it is essential to consider that in regions with a high endemicity of TB, secondary prophylaxis may be warranted.

## Conclusions

TB and HIV constitute formidable challenges in public healthcare globally. Our understanding of the coinfection of these two diseases has advanced significantly, and we are now well aware of the mechanisms that heighten the vulnerability of HIV-infected individuals to TB. Despite the availability of effective antibiotics, TB continues to be a major threat to public health, particularly in developing countries. In individuals with intact immune systems, TB bacilli are often contained within granulomas, resulting in LTBI. However, when the host's immune system weakens, latent TB can become active, leading to increased bacterial numbers and disease progression. This transition results in clinical symptoms and TB transmission. Standard drug-sensitive TB infections can be managed with first-line anti-TB medications, but DR-TB poses more significant challenges and yields less favorable outcomes, contributing to the enduring TB pandemic. Efforts to control TB are impeded by factors such as HIV coinfection, the ongoing COVID-19 pandemic, patient adherence issues, and inadequate treatment strategies in various regions across the globe. These challenges underscore the importance of continued research and a multifaceted approach to address the complex interactions between TB and HIV.
